# Molecular Imaging of Aortic Valve Stenosis with Positron Emission Tomography

**DOI:** 10.3390/ph15070812

**Published:** 2022-06-30

**Authors:** Reindert F. Oostveen, Yannick Kaiser, Erik S.G. Stroes, Hein J. Verberne

**Affiliations:** 1 Department of Vascular Medicine, Amsterdam Cardiovascular Sciences, Amsterdam UMC, University of Amsterdam, 1105 AZ Amsterdam, The Netherlands; r.oostveen@amsterdamumc.nl (R.F.O.); y.kaiser@amsterdamumc.nl (Y.K.); e.s.stroes@amsterdamumc.nl (E.S.G.S.); 2 Department of Radiology and Nuclear Medicine, Amsterdam UMC, University of Amsterdam, 1105 AZ Amsterdam, The Netherlands

**Keywords:** aortic valve stenosis, positron emission tomography, fluorodeoxyglucose, sodium fluoride

## Abstract

Aortic valve stenosis (AVS) is an increasingly prevalent disease in our aging population. Although multiple risk factors for AVS have been elucidated, medical therapies capable of slowing down disease progression remain unavailable. Molecular imaging technologies are opening up avenues for the non-invasive assessment of disease progression, allowing the assessment of (early) medical interventions. This review will focus on the role of positron emission tomography of the aortic valve with ^18^F-fluorodeoxyglucose and ^18^F-sodium fluoride but will also shed light on novel tracers which have potential in AVS, ranging from the healthy aortic valve to end-stage valvular disease.

## 1. Relevance

Aortic valve stenosis (AVS) is the world’s most common valvular heart disease.

In 2019, a total of 9.4 million individuals suffered from AVS in the United States of America, a 443% increase compared to the 1990 prevalence [1]. Roughly 3.4% of individuals aged 75 and over suffer from AVS, and the total prevalence is expected to have tripled once again by 2050, mainly due to the aging population, in which increasing numbers of individuals are living with chronic cardiovascular diseases [2]. The age-dependent nature of AVS was further emphasized in a study with 11,911 adults undergoing echocardiography in Minnesota. This study concluded that the prevalence of moderate to severe AVS increased from a mere 0.02–0.1% in patients aged 18 to 44 years to 2.8–4.6% in patients over the age of 75 [3]. 

Currently, the only treatments for severe AVS are surgical or transcatheter aortic valve replacement, which are costly procedures that are not suitable for all patients or even feasible in all regions of the world. These procedures are also accompanied by periprocedural morbidity and mortality, especially in an older population with significant co-morbidities [4]. Therefore, the need for (preventative) medical interventions in aortic valve stenosis is immense. Attempts have been made to find effective medical interventions to prevent AVS, but so far, they have remained unsuccessful. Two statin trials failed to reduce AVS disease progression; nonetheless, hopes are still vested in apolipoprotein-B-containing lipoproteins providing the key for the prevention of aortic valve stenosis [5,6]. This is because the PCSK9 R46L loss-of-function mutations, which cause reduced exposure to these lipoproteins, are associated with a reduced risk of aortic valve stenosis [7]. Data from a murine study has also hinted that PCSK9 is a facilitator of calcification in valvular interstitial cells [8]. However, PCSK9 inhibition therapy to reduce aortic valve stenosis progression is still to be researched. So, although AVS is characterized by a slow disease progress that can span decades, as of yet, no medical therapies are available to attenuate disease progression [9]. This may partially be attributed to the fact that conventional imaging methodologies to assess AVS progression—echocardiography and computed tomography—are incapable of detecting disease stages prior to the onset of calcification [10]. In this review, we will discuss advances in positron emission tomography of the aortic valve with ^18^F-fluorodeoxyglucose (^18^F-FDG) and ^18^F-sodium fluoride (^18^F-NaF) and shed light on novel molecular tracers that may be of use in the future to identify aortic valve stenosis at an earlier, pre-calcified stage.

## 2. Pathophysiology

AVS can be caused by rheumatic disease and congenital malformations of the aortic valve, such as unicuspid and (more commonly) bicuspid valves. However, in the majority of cases it arises due to the calcification of a normal tri-leaflet valve.

For a long time, AVS was considered a degenerative disease, a condition wherein years of mechanical stress were the primary cause of the progressive accumulation of calcium deposits within the valve. However, AVS is now increasingly recognized as an active disease process, which is driven by a multitude of inflammatory and pro-osteogenic cascades, sharing many features with the pathophysiology of atherosclerosis. The current belief is that this more complex active disease process can be split into two relatively distinct disease phases: the initiation phase and the propagation phase [11].

### 2.1. Initiation Phase

The initiation phase is “initiated” by mechanical stressors, which induce damage to the endothelial cell layer of the valve. The importance of these mechanical stressors in the pathology of AVS is highlighted in patients with bicuspid valves. It was shown that in patients who underwent aortic valve replacement for AVS and were over the age of 80, 20% had bicuspid aortic valves, compared to over 50% of the younger patients between 60 and 80 years old [12].

The damage caused to the endothelial layer of the aortic valve allows for lipids such as lipoprotein(a) and low-density lipoprotein (LDL) and immune cells such as macrophages, T lymphocytes, and mast cells to enter the inner layers of the aortic valve, which comprises valvular interstitial cells (VICs) [13]. Within these inner layers, the lipoproteins undergo oxidative modification [14,15], which ignites an inflammatory reaction very similar to the inflammatory response seen in atherosclerosis.

The VICs are directly activated by the cytotoxic oxidized LDL through toll-like receptors 2 and 4 and, as a result, proliferate and undergo osteogenic transformation [16]. The activated VICs will remodel the extracellular matrix through the production of unorganized collagen, cathepsins, and metalloproteinases [17,18]. After osteogenic transformation, these osteoblast-like cells will also produce and deposit calcified vesicles, causing further thickening and stiffening of the aortic valve.

### 2.2. Propagation Phase

The formation of calcium deposits subsequently leads to the onset of the propagation phase, in which calcium begets calcium by inflicting further damage to the valve. During the propagation phase, disease progression is accelerated; once severe calcifications have developed, AVS progression may even be completely independent from initiating risk factors [19].

To adequately assess the effect of medical therapies, imaging tools capable of assessing disease progression in different AVS stages are required.

## 3. Risk Factors for AVS

The known risk factors for AVS generally overlap with those for atherosclerosis and include increasing age, male sex, smoking, hypercholesterolaemia, hypertension, elevated lipoprotein(a), diabetes mellitus, kidney failure, and aberrant aortic valve morphology [20]. In contrast, LDL-cholesterol lowering with statins was shown in two separate double-blind placebo-controlled trials to be unable to attenuate disease progression in patients with established AVS [5,6]. This may be due to the difference between initiating and propagating factors in AVS, stressing the importance of imaging modalities that are able to assess not only the propagation but also the initiation of AVS.

## 4. Conventional Imaging Modalities

Classic clinical manifestations of AVS, such as heart failure, syncope, and angina, typically do not occur prior to the development of severe AVS. As a result, AVS is often an incidental finding due to the presence of a systolic murmur or is detected by echocardiography performed for other indications [10].

Echocardiography is currently the main method for evaluating the severity of AVS. To this end, it uses three parameters: the aortic valve area, the mean pressure gradient across the aortic valve, and the peak velocity of forward flow across the diseased valve. Severe AVS is defined by an aortic valve area < 1 cm², a mean pressure gradient of >40 mmHg, and a peak velocity of >4 m/s. In a straight-forward, ideal scenario, these three parameters are concordant. Unfortunately, despite precise methodology, between 20% and 30% of echocardiography procedures still yield discordant results, wherein, for example, the aortic valve area is <1 cm² but the mean pressure gradient is <40 mmHg. These discordant results create uncertainties regarding outcomes and incidence.

In the case of discordant echocardiography parameters, computed tomography (CT) imaging may be performed to assess disease severity [21]. Calcium scoring using CT has been shown to be highly correlated with the haemodynamic severity observed via echocardiography [22]. The latest version of the ESC/EACTS guidelines on valvular heart disease states that in the case of discordant grading through echocardiography, calcium scoring should be conducted using CT [23]. A recent meta-analysis showed that the aortic valve calcium score (AVCS), determined by CT, demonstrated moderate sensitivity and specificity (82% and 78%, respectively) for diagnosing severe AVS. The AVCS was also shown to be associated with higher rates of all-cause mortality [24]. Echocardiography and CT imaging have become established imaging techniques for assessing the severity of AVS. However, an important limitation of these imaging modalities is that aortic valve disease typically remains undetectable until significant calcifications have developed. Therefore, these imaging techniques are unable to assess the effect of treatment in earlier stages of aortic valve disease.

## 5. Molecular Imaging of AVS

### 5.1. ^18^F-Fluorodeoxyglucose (^18^F-FDG)

^18^F-FDG is the most commonly used PET tracer. ^18^F-FDG is structurally very similar to glucose, and therefore cells that metabolize glucose will also absorb ^18^F-FDG using glucose transporters 1 and 3. A cytosolic enzyme, hexokinase, then phosphorylates ^18^F-FDG as it would glucose. However, ^18^F-FDG is not capable of entering the glycolysis pathway and therefore accumulates within the cell [25]. Therefore, ^18^F-FDG uptake detected by PET/CTs is a representation of the metabolic activity within tissue. ^18^F-FDG is primarily taken up in metabolically active tissue such as the brain, the myocardium, and cancer cells; the latter being the most common clinical indication for performing ^18^F-FDG imaging. ^18^F-FDG uptake is also increased in inflamed tissue and is therefore used as a diagnostic tool in patients with a fever of unknown origin. Furthermore, in patients with suspected infective endocarditis and with prosthetic heart valves, ^18^F-FDG has incremental diagnostic value [26].

The incidental observation that patients with atherosclerosis often demonstrated high uptake in the aorta and carotid arteries led to studies using ^18^F-FDG to assess arterial wall inflammation. Rudd et al. were the first to study whether ^18^F-FDG uptake in a symptomatic carotid plaque differed compared to a contralateral asymptomatic lesion [27]. Sixty patients with a recent stroke, transient ischemic attack, or retinal embolism were included. This study showed that ^18^F-FDG uptake was greater in the ipsilateral carotid plaques in patients with an early recurrent stroke. Additionally, the accumulation of ^18^F-FDG on the autoradiograph was mainly co-localized with macrophage-rich areas of the plaque. Later studies demonstrated that ^18^F-FDG uptake in carotid arteries improves cardiovascular risk prediction on top of the Framingham score [28]. The high reproducibility of assessing carotid ^18^F-FDG uptake led to PET imaging being used as a surrogate outcome in several randomized trials [29]. For instance, cholesterol lowering by a 12-week intensification of statin therapy led to a significant reduction in carotid ^18^F-FDG uptake, in line with the beneficial effect of statins on cardiovascular outcomes in randomized trials [30]. Marincheva-Savcheva et al. extended these observations to AVS, given the similarities between the two disease processes [31]. Eighty-four patients who had undergone PET/CTs for the evaluation of a neoplastic process and had an echocardiographically confirmed diagnosis of AVS were retrospectively identified. Compared to the control group, patients with AVS had significantly higher uptakes of ^18^F-FDG as expressed by the target-to-background ratio (TBR). It was postulated that this increased uptake of ^18^F-FDG represents the inflammatory activity within the aortic valve, since other cells located within the aortic valve are not very likely to show an increase in glucose metabolism. Interestingly, patients with severe AVS had comparable ^18^F-FDG uptake to control subjects. This could be evidence of another overlap between the pathophysiology of AVS and atherosclerosis, since studies have shown that in calcified plaques, macrophage content is reduced when compared to early plaques [32,33]. A subsequent prospective imaging study by the same group showed that in a population of 1410 patients, a higher ^18^F-FDG PET/CT signal at baseline without established aortic stenosis was associated with an increased likelihood of future valvular calcification [34]. Dweck and colleagues observed a further increase in valvular ^18^F-FDG uptake in patients with severe AVS compared to patients with mild to moderate AVS, albeit a modest one [35]. As the largest relative increase in valvular ^18^F-FDG is demonstrated between healthy control subjects and those with moderate AVS, ^18^F-FDG imaging may be particularly suitable for assessing the effect of treatment in the early disease stages of AVS (especially in combination with ^18^F-sodium fluoride, to be discussed below).

However, in contrast to arterial wall inflammation, the reversibility of valvular ^18^F-FDG uptake has not been previously assessed. Accordingly, trials investigating the effect of lipid-lowering therapy using either statins, PCSK9 inhibitors, or apo(a) antisense on valvular ^18^F-FDG uptake in patients at increased risk of AVS are warranted.

### 5.2. ^18^F-sodium fluoride (^18^F-NaF)

^18^F-NaF has been used clinically as a bone tracer for over half a century, for example, as a diagnostic instrument in Paget’s disease [36]. In the past decade, its use has been extended to include the assessment of vascular and valvular calcifications [37,38,39,40,41]. The mechanism of action of ^18^F-NaF is dependent on hydroxyapatite [42]. ^18^F-NaF binds to hydroxyapatite crystals, exchanges its ^18^F-fluoride ion with the hydroxyl groups on hydroxyapatite, and forms fluoroapatite, which is thereafter incorporated within the bone-matrix or calcified tissue. ^18^F-NaF uptake therefore reflects osteoblastic activity, a key feature of AVS. PET/CTs using ^18^F-NaF can be of greater value than echocardiography and CT, since these imaging techniques merely provide information on already established calcification, whilst ^18^F-NaF PET/CTs can offer information about the ongoing calcification activity.

Dweck et al. were the first to evaluate ^18^F-NaF as a marker of calcification activity in AVS [35]. They have shown that baseline valvular ^18^F-NaF uptake is highly correlated with the progression of the aortic valve calcium score assessed via CT after 1 year (r = 0.75), whereas ^18^F-FDG uptake is not. Additionally, valvular ^18^F-NaF correlated with alkaline phosphatase and osteocalcin immunohistochemistry, lending further support to ^18^F-NaF as a marker of calcification activity. In the same study, they compared ^18^F-NaF with ^18^F-FDG uptake in different stages of AVS and showed that valvular ^18^F-NaF uptake increased more pronouncedly with increasing disease severity than ^18^F-FDG. These observations led to ^18^F-NaF being used to assess whether newly identified risk factors for AVS are associated with valvular ^18^F-NaF uptake as a marker of calcification activity, and thus of progressive disease. Zheng et al. performed a post hoc analysis of two longitudinal imaging studies (Scottish Aortic Stenosis and Lipid Lowering Trial, Impact on Regression (SALTIRE) and Ring of Fire, with PET available for 81 patients from the Ring of Fire study), showing that participants in the upper Lp(a) tertile (>35 mg/dL) had increased valvular ^18^F-NaF uptake, which corresponded with accelerated AVS progression as measured by echocardiography and CT, ultimately leading to more valvular replacement [43]. Deprès et al. later found similarly increased ^18^F-NaF uptake in patients with elevated Lp(a) who were free from aortic valve disease [44]. In contrast, in a case-control study by our group, in which we matched individuals with elevated Lp(a) (>50 mg/dL) to those with low Lp(a), we observed no elevated ^18^F-NaF uptake in patients with high Lp(a) after careful matching for AVS disease severity [45]. All of the included subjects had at least mild to moderate AVS. A limitation of this study was that patients in the low Lp(a) group had significantly higher blood pressure and low-density-lipoprotein cholesterol levels, which may have contributed to similar ^18^F-NaF uptake in both groups. To date, there has been one randomized clinical trial employing PET imaging of the aortic valve, with several more currently ongoing. The Study Investigating the Effect of Drugs Used to Treat Osteoporosis on the Progression of Calcific Aortic Stenosis (SALTIRE II) investigated whether denosumab or alendronic acid affect ^18^F-NaF uptake and/or the progression of CT- and echocardiography-assessed AVS severity [46]. Unfortunately, neither denosumab nor alendronic acid affected ^18^F-NaF uptake or the progression measured by CT or echocardiography. Ongoing trials are investigating the effect of PCSK9 (NCT03051360) and vitamin K2 (NCT02917525) in AVS patients.

## 6. Future Tracers

^18^F-FDG and ^18^F-NaF have so far yielded the most promising results, as discussed above. However, other tracers are available that have potential in AVS and warrant more research.

### 6.1. ^68^Ga-Dotatate

^68^Ga-Dotatate is a PET marker which binds specifically to somatostatin receptor type 2 (SSTR2) [47]. It has been used for over a decade to diagnose rare neuroendocrine tumors, since this neoplasm commonly has a high expression of SSTR2 [48]. SSTR2 has also been shown to be highly expressed on classical (M1-activated) macrophages, which are abundant in plaques predicting an unfavorable phenotype, making ^68^Ga-Dotatate a suitable marker for vascular wall inflammation [49]. Incidental findings on diagnostic scans for patients suspected of having neuroendocrine tumors showed significant ^68^Ga-Dotatate uptake within aortic plaques, which prompted research using this tracer as a marker for vascular wall inflammation. Tarkin et al. published results from the autoradiography of eight plaques that confirmed high levels of specific Dotatate ligand binding to SSTR2 [49]. They also observed strong SSTR2 and CD68 mRNA correlation, which in turn also strongly correlated with ^68^Ga-Dotatate uptake, further validating ^68^Ga-Dotatate as a macrophage-specific marker. In addition, the authors observed a strong co-localization of SSTR2 and CD68, as assessed with immune histology.

Recent studies have therefore repurposed ^68^Ga-Dotatate PET/CT to identify vascular wall inflammation in the carotids, coronaries, and aorta and have demonstrated its superiority to the traditionally used ^18^F-FDG tracer. This is mainly due to the fact that ^68^Ga-Dotatate is not affected by glucose levels and does not suffer from the same physiological myocardial uptake as ^18^F-FDG, which leads to spillover that may render over 50% of coronary ^18^F-FDG scans uninterpretable. Assessing ^18^F-FDG in the aortic valve is not hindered as drastically by myocardial spillover as in the coronaries; however, the specificity of ^68^Ga-Dotatate and the fact that it is not influenced by glucose levels are still significant advantages.

To our knowledge, no studies have investigated ^68^Ga-Dotatate as a tracer for AVS. However, its overlapping pathophysiology with atherosclerosis, the results we have seen using a different marker of inflammation (^18^F-FDG), and the benefits of ^68^Ga-Dotatate when compared to ^18^F-FDG in regard to specificity and usability make us believe that this tracer deserves more consideration in the field of AVS.

### 6.2. ^18^F-GP1

^18^F-GP1 is a novel tracer that has shown promising results, particularly in bioprosthetic valves. ^18^F-GP1 binds to the glycoprotein IIb/IIIa receptor, which is expressed on activated platelets [50]. It has been shown to detect both arterial and venous thrombi in vivo.

One study that examined 53 bioprosthetic aortic valves and 22 native tri-leaflet aortic valves demonstrated the uptake of ^18^F-GP1 in all of the bioprosthetic valves but in none of the native valves [51]. This increased uptake of ^18^F-GP1 was associated independently with the age of the bioprosthetic valve and with hypo attenuated leaflet thickening. Three participants had obstructive valve thrombosis, and after anticoagulation therapy reductions in ^18^F-GP1 uptake were observed, demonstrating the potential of ^18^F-GP1 as a clinical tool.

## 7. Limitations

The molecular imaging of the aortic valve using PET has several limitations that deserve to be addressed. First, a fundamental limit of PET is its spatial and temporal resolution, which hampers its use for assessing tracer uptake in small and moving structures such as the aortic valve [52]. Although the temporal resolution of myocardial PET imaging can be significantly improved by using an ECG trigger during the image acquisition, the spatial resolution is still far behind that obtained with other imaging techniques such as CT or MRI.

Second, although the reversibility of ^18^F-FDG uptake has previously been shown in the carotid arteries, no such studies exist for the aortic valve, as the high myocardial background uptake of ^18^F-FDG limits its utility in assessing coronary and valvular uptake [50]. For ^18^F-NaF, no reversibility has previously been demonstrated in the aortic valve or the arterial wall, in line with the absence of medical interventions capable of attenuating calcification. Lastly, it is important to note that whilst ^18^F-NaF uptake is strongly correlated with the progression of calcium, it does not predict subsequent calcification independent of the amount of baseline calcium present.

## 8. Conclusions

The molecular imaging of the aortic valve with PET is a promising method for assessing novel treatment strategies that may attenuate AVS progression. However, significant limitations remain. Although ^18^F-NaF uptake in the valve correlates particularly well with disease progression, it has not been shown to improve the prediction of progressive calcification over only baseline calcium scores, and reversibility has yet to be shown. Whereas reversibility in arterial wall inflammation has been demonstrated for ^18^F-FDG, this characteristic has yet to be validated in the aortic valve.

## Data Availability

Data sharing not applicable.
